# Assessing veterinary education outcomes in livestock systems: a quasi-experimental study of scientific buffalo husbandry training

**DOI:** 10.3389/fvets.2025.1656517

**Published:** 2025-08-26

**Authors:** Gururaj Makarabbi, Aiswarya Sabu, Navneet Saxena, Madan Lal Sharma

**Affiliations:** Central Institute for Research on Buffaloes (ICAR), Hisar, India

**Keywords:** scientific buffalo husbandry, cognitive gain, training effectiveness, knowledge test, data-driven extension

## Abstract

Scientific buffalo husbandry management practices play a pivotal role in enhancing rural livelihoods and milk productivity in India. Despite widespread training initiatives, there is limited empirical evidence quantifying the cognitive impact of such programs on smallholder farmers. This study, grounded in Adult Learning Theory (Andragogy), adopted a quasi-experimental one-group pre-test–post-test design involving 518 farmers trained at the Indian Council of Agricultural Research—Central Institute for Research on Buffaloes (ICAR-CIRB). A validated 15-item knowledge test was used to assess learning across five thematic domains: breeding, feeding and nutrition, animal health, milk quality, and milk marketing. The training intervention resulted in a substantial improvement in farmers' knowledge, as evidenced by both absolute and normalized learning gains. Subject-wise analysis revealed feeding and nutrition as the most improved domain, followed by milk marketing and breeding. Multiple linear regression analysis identified specific socio-demographic and experiential factors—such as gender, livestock holding, and prior experience—as significant predictors of knowledge gain, while age and baseline knowledge showed negative associations. Disaggregated profiling further showed that young, moderately experienced female farmers with medium-to-large herd sizes achieved the highest learning gains. These findings suggest that training outcomes vary considerably across learner profiles and subject areas. The study highlights the importance of targeted, learner-sensitive extension models not only for enhancing knowledge transfer, but also for promoting the adoption of best practices, and for enabling systematic monitoring and evaluation of how the trained subject matter areas are implemented at the field level.

## 1 Introduction

**Figure 1:**
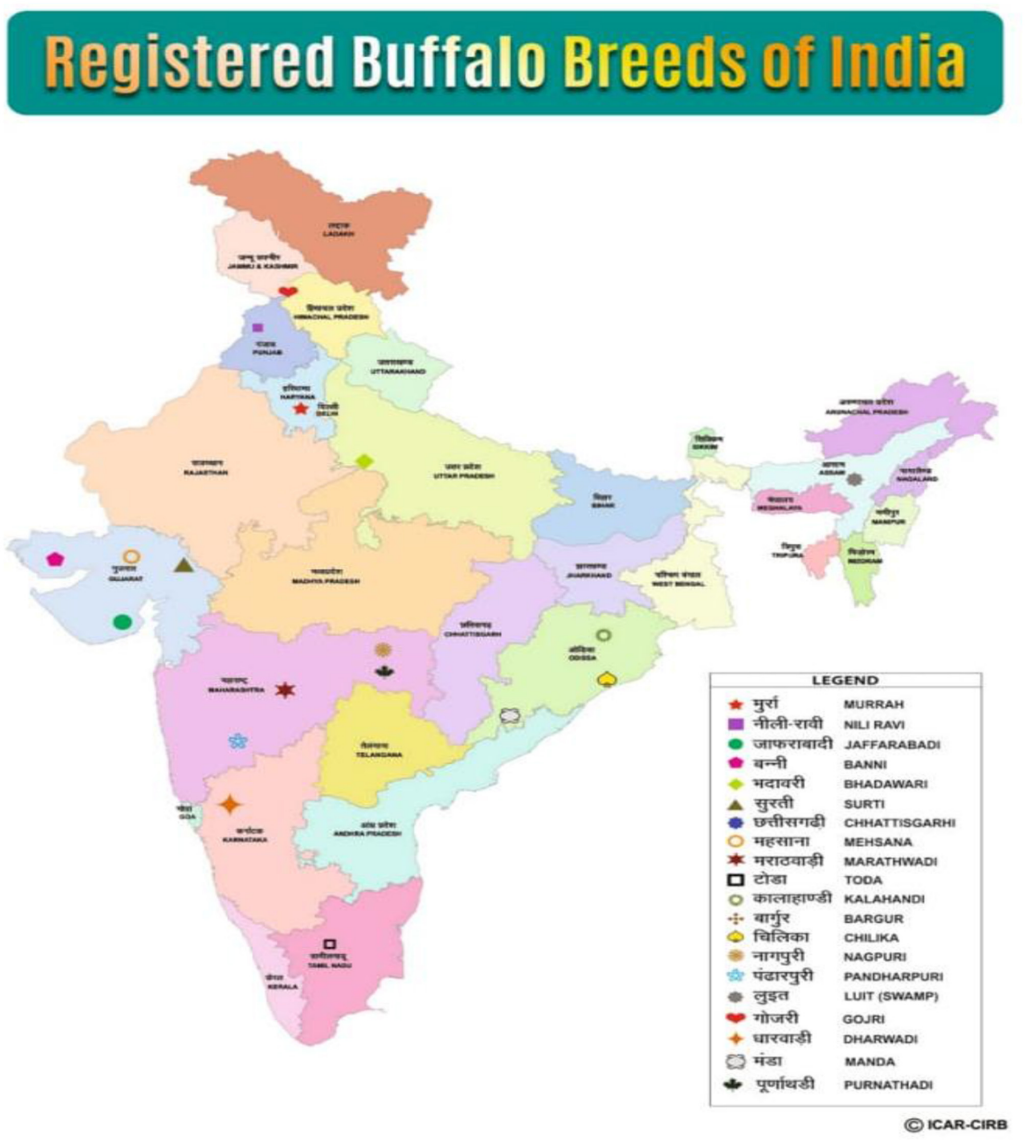
Registered buffalo breeds of India.

Veterinary science is central to the sustainable management of livestock systems, especially in terms of animal health, biosecurity, production efficiency, and One Health integration (1). Buffalo husbandry is integral to India's livestock economy, with approximately 109.85 million buffaloes representing 56.8% of the world's buffalo population (1). These animals contribute more than 100 million tons of milk annually, accounting for 45% of the nation's milk output and ^₹^1.5 trillion in economic value—~7.4% of India's agricultural GDP (1, 2). In addition to their milk yield and high butterfat content, buffaloes present unique veterinary attributes including stronger immunity to hemoprotozoan infections, better adaptation to endemic parasitic loads, and physiological resilience under thermal stress—making them highly relevant to disease control, veterinary epidemiology, and herd health planning (3). As of June 2025, a total of 20 buffalo breeds have been officially registered in India by the ICAR–National Bureau of Animal Genetic Resources (NBAGR). These breeds are distributed across five major geographic zones as shown in Figure 1: Northern (Murrah, Nili-Ravi, Gojri), Western (Jaffarabadi, Mehsana, Surti, Banni), Central (Bhadawari, Chhattisgarhi, Nagpuri, Marathwadi, Pandharpuri), Eastern (Chilika, Kalahandi, Manda, Luit), and Southern (Toda, Bargur, Dharwadi). The Murrah breed, extensively reared in Haryana and Punjab, is regarded as the most notable due to its superior milk productivity, rich fat content, and ability to thrive in a range of agro-climatic conditions (4).

The socioeconomic relevance of buffalo husbandry is amplified by its disproportionate reliance on smallholder farmers, who constitute over 70 million households (5). Rural women, in particular, perform 85% of routine buffalo management tasks (6), thereby making the sector an important conduit for achieving Sustainable Development Goals such as SDG 1 (No Poverty), SDG 2 (Zero Hunger), and SDG 5 (Gender Equality). However, despite its potential, the adoption of scientific husbandry practices—such as artificial insemination, balanced nutrition, mineral mixture supplementation, and evidence-based veterinary interventions like mastitis control, deworming schedules, vaccination protocols, and ethno-veterinary integration—remains strikingly low (7, 8). This adoption lag undermines both productivity and welfare gains. Numerous studies have established that practices like mineral supplementation and Artificial Insemination can significantly improve milk yield (by 42–58%) and reduce environmental footprints (9), while hygienic milking improves quality and revenue (10). Yet, the persistent Knowledge–Attitude–Practice (KAP) gap indicates that the mere availability of technology is insufficient (11, 12). The core issue lies in inadequate knowledge dissemination and low cognitive engagement among farmers (13). Deficiencies in veterinary outreach, weak last-mile connectivity of animal health workers, and low engagement with participatory extension models hinder effective veterinary communication and health literacy among farmers (14, 15).

In the veterinary domain, capacity building is not only about knowledge transfer—it underpins preventive animal health, zoonotic disease surveillance, antimicrobial stewardship, and sustainable food systems. Evaluating training outcomes in veterinary education is therefore critical (16). In India, where livestock—particularly buffaloes—play a critical role in rural livelihoods and climate resilience, assessing the effectiveness of veterinary training efforts across key domains such as reproductive management, disease diagnostics, and hygiene compliance is essential. Buffaloes, due to their multifunctional role in smallholder production systems and their prominence in India's dairy value chain, are of particular interest to veterinary public health frameworks (17, 18). Training programmes must therefore be evaluated using robust veterinary indicators—such as improved recognition of disease symptoms, adherence to vaccination timetables, mastitis detection skills, and knowledge of withdrawal periods for antibiotics and anthelmintics—rather than mere attendance or satisfaction scores. Despite significant institutional investments in livestock training, empirical evaluations of knowledge acquisition remain limited, weakening efforts to foster meaningful behavioral change and long-term adoption of scientific practices. Existing research tends to focus on adoption outcomes or productivity metrics, often overlooking the vital cognitive transformations in farmers that serve as precursors to veterinary compliance and biosecurity (19–23). Moreover, psychological, educational, and institutional barriers—such as entrenched traditional norms, low formal education, and mistrust—continue to hinder effective knowledge assimilation (15). This study aims to bridge these gaps by measuring the cognitive impact of training and identifying the socio-economic factors that influence knowledge gains, ultimately contributing to more accountable and targeted livestock extension strategies.

Against this backdrop, the current study proposes a structured framework to evaluate the cognitive impact of scientific buffalo husbandry training delivered to smallholder farmers. This research applies a domain-specific knowledge test to quantify learning outcomes before and after the intervention by employing a quasi-experimental one-group pre-test–post-test design. The study explores four major domains: breeding, feeding, healthcare, and hygienic management, while simultaneously assessing the influence of socio-demographic variables such as age, education, herd size, and landholding. This approach provides a comprehensive understanding of how training translates into knowledge enhancement and which socio-economic segments benefit the most. Ultimately, the study aims to offer actionable insights for the design of more effective, targeted, and evidence-based veterinary extension and animal health education programmes that support public health, productivity, and welfare goals. The specific objectives of the study are as follows:

- To measure the cognitive gain of farmers resulting from structured training in scientific buffalo husbandry practices.
- To compare knowledge improvement across core domains—breeding, nutrition, healthcare, and hygienic practices—among trained farmers.
- To examine the influence of socio-economic characteristics on the extent of cognitive gain among smallholder buffalo rearers.

The following hypotheses were tested under the present study:

H1: Structured training on scientific buffalo husbandry significantly improves farmers' cognitive knowledge scores from pre-test to post-test.
H2: The cognitive gain resulting from training varies significantly across the core domains of buffalo husbandry—breeding, nutrition, healthcare, and hygienic practices.
H3: Socio-economic characteristics of farmers (such as education, age, herd size, and landholding) significantly influence the extent of cognitive gain achieved through training.

## 2 Theoretical foundation

**Figure 2:**
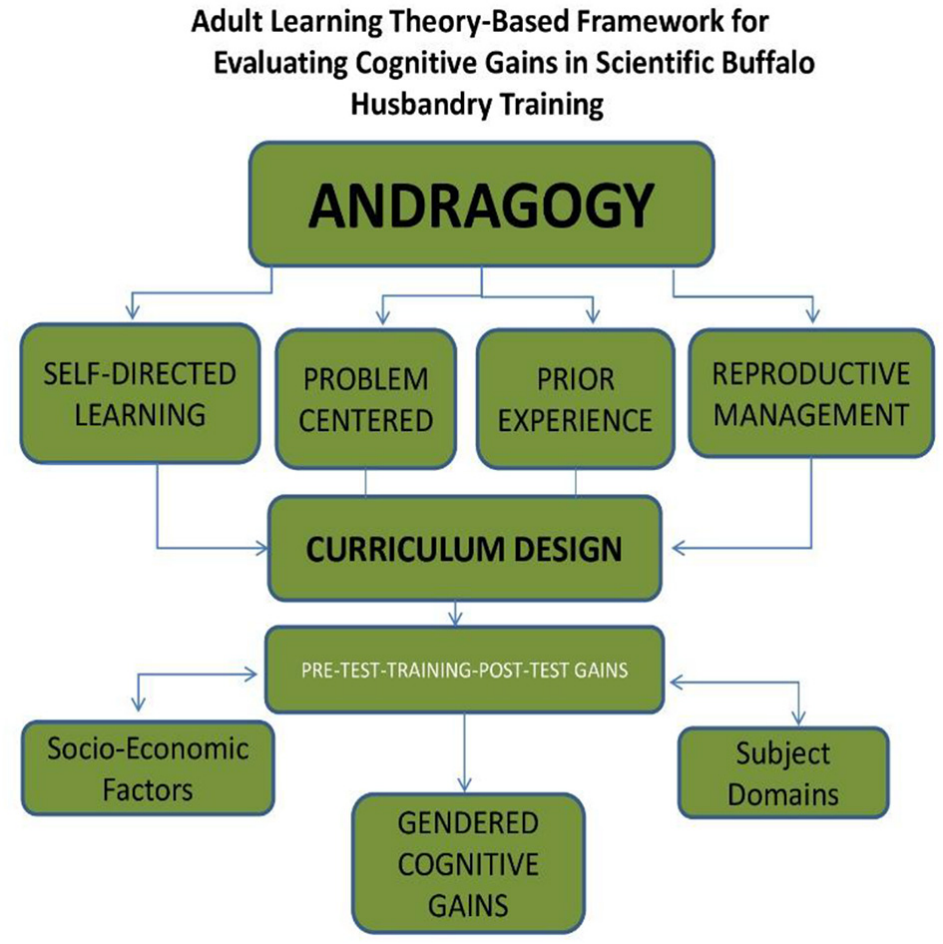
Theoretical foundation of the study.

This study is grounded in Adult Learning Theory (Andragogy), as conceptualized by Knowles (24), which asserts that adult learners are self-directed, purpose-driven, and bring a wealth of prior experience that shapes how they acquire and apply new knowledge (Figure 2). Knowles emphasized that adults learn most effectively when the content is relevant to their immediate needs, problem-oriented, and allows for autonomy in the learning process. In this context, the target group—smallholder buffalo farmers—are adult learners with deep experiential knowledge of livestock management but often lack formal exposure to scientific veterinary practices (13, 15). Recognizing the lack of scientific knowledge, the training curriculum was thoughtfully designed in line with andragogical principles. The modules were developed to be practical, directly applicable, and tailored to address specific challenges faced by farmers in the domains of breeding, nutrition, healthcare, and hygienic practices. This approach ensured that the training was not only contextually meaningful but also respectful of learners' autonomy and intrinsic motivation (25, 26). The use of a domain-specific knowledge test administered before and after training also reflects the theoretical foundation. It provided a structured mechanism to assess cognitive gains while acknowledging the adult learner's preference for measurable progress tied to real-life applications. By focusing on concept clarity and knowledge retention in areas critical to animal health, this tool allowed for a more authentic evaluation of learning outcomes. In addition, the study explored how socio-economic factors such as age, education, herd size, and landholding influenced learning gains. This examination of contextual influences on adult learning ascertain that adult learning is heavily shaped by personal background, life context, and perceived utility of knowledge (25, 26). In operationalizing these principles, the study recognizes that adult education—particularly in rural, resource-constrained settings—is most effective when it adapts to the learner's environment and leverages their prior experiences. The study contributes to a theory-driven and evidence-based model for veterinary education, particularly in the context of rural livestock systems. By integrating andragogical principles with empirical evaluation, the study offers a replicable framework for designing and assessing capacity-building interventions that are both scientifically rigorous and learner-centered.

## 3 Materials and methods

### 3.1 Research design

The present study adopted a quasi-experimental one-group pre-test–post-test design to evaluate the cognitive gain of smallholder buffalo farmers who participated in a structured capacity-building programme. This design enabled the measurement of knowledge improvement attributable to the training by comparing participants' knowledge scores before and after the intervention.

### 3.2 Study area and sample description

The study was conducted at the ICAR-Central Institute for Research on Buffaloes (CIRB), Hisar, a premier national institute under the Indian Council of Agricultural Research (ICAR), Ministry of Agriculture and Farmers' Welfare, Government of India. CIRB plays a pivotal role in advancing scientific buffalo husbandry through its core mandate of research, breed improvement, and capacity-building initiatives targeting stakeholders across India. Data for the present study were collected from farmers who participated in the on-campus training programs on Scientific Buffalo Husbandry Practices, regularly organized by CIRB. These structured training sessions are part of the institute's flagship knowledge dissemination mandate and attract participants from multiple states across India. The training is conducted monthly, with each batch comprising approximately 30 farmers, and is designed to enhance technical competencies in buffalo breeding, feeding and nutrition, animal health management, milk marketing, and value addition. The training program is consistently delivered over a structured duration of five consecutive days, combining both theoretical sessions and hands-on practical exposure on the institute's model buffalo farms and laboratories.

### 3.3 Sampling framework and selection criteria

The study employed a purposive sampling technique to select participants from among those enrolled in the CIRB training programmes conducted between January 2023 and December 2024. A total of 530 participants were selected based on the following inclusion criteria:

1. The participant must own at least one buffalo at the time of training.
2. The participant must have a minimum of 1 year of buffalo rearing experience.
3. The participant must be actively involved in buffalo farming as a primary livelihood activity.

Out of the total 1,120 farmers who attended the training, a substantial number were excluded from the final sample. Exclusion was due to various reasons, including incomplete responses, absence during either the pre- or post-training assessments, and challenges in comprehension of the assessment tools, particularly among illiterate participants. While the study does not claim statistical representativeness of the entire buffalo-farming population, the final sample was intentionally selected to reflect diversity in farm sizes, management practices, and production systems.

### 3.4 Instrumentation and validation

#### 3.4.1 Development of knowledge test

Sullivan and Dunton (27) outlined a systematic process for knowledge test development, encompassing the generation of pilot items, expert review, and evaluation of item properties such as difficulty and discrimination (27). Following this rigorous framework, a structured knowledge test was developed to measure the cognitive gain of farmers participating in training on scientific buffalo husbandry practices organized by ICAR-CIRB. The test construction began with the identification of five core thematic areas relevant to the training content: (1) Feeding, (2) Breeding, (3) Health Management, (4) Milk Quality, and (5) Milk Marketing. From each domain, five items were meticulously formulated, resulting in an initial pool of 25 items. Each item was directly mapped to specific content delivered in the lecture sessions. For instance, the item on dry matter percentage in buffalo feed (F1) reflected the content discussed during the lecture on Nutrient Requirements and Ration Balancing, while mineral mixture frequency (F2) was covered in the session on Feeding Strategies for High-Yielding Buffaloes. The question on optimal AI timing (B1) aligned with the lecture titled Reproductive Efficiency and Heat Detection Techniques, and HS+BQ vaccination (H1) was based on content from Buffalo Disease Management and Preventive Health Strategies. The milk quality question on SNF values (MQ1) was taken from the session Clean Milk Production and Quality Parameters, and milk marketing (MM1) referred to content from the lecture Milk Marketing Channels and Cooperative Models.

All items were designed to assess factual knowledge and conceptual understanding and were framed in either multiple-choice or true/false formats. A panel of 11 experts, including buffalo husbandry scientists, extension professionals, and training coordinators from CIRB, reviewed the initial item pool. Using predefined criteria—clarity of language, scientific accuracy, relevance to buffalo rearing practices, appropriateness to the literacy level of smallholder dairy farmers, and alignment with the training curriculum—the panel provided feedback for refinement. Based on their judgment, necessary revisions were made to enhance content validity. The revised 25-item test was then administered to a separate group of 120 non-sample farmers—distinct from those participating in the main study. This group was selected to evaluate the psychometric properties of the test without influencing the study sample. Data from this pre-test were used to compute item difficulty indices, item discrimination indices, and overall test reliability using Cronbach's alpha. Each correct response was scored as 1, and incorrect or non-responses were scored as 0, with a maximum possible score of 25. The final validated tool was administered to participants as both a pre-test and post-test to assess knowledge improvement following the training.

##### 3.4.1.1 Item difficulty index

Also known as the *p*-value, the difficulty index indicates the proportion of respondents who correctly answered a given item. Correct responses were coded as 1, and incorrect responses as 0. A high *p*-value implies the item was easy, while a very low value suggests that the item was difficult.

The formula used for calculating the difficulty index was: pi = (Number of respondents who answered the item correctly)/(Total number of respondents).

In the present study, difficulty index values ranged from 0.18 to 0.91 as given in Table 1, covering a wide spectrum of item easiness. An ideal knowledge test should include a good mix of items with difficulty indices ranging from 0.30 to 0.85 (27). Items with *p*-values above 0.90 are considered too easy, and those below 0.30 are generally considered too difficult and may fail to contribute meaningfully to measurement precision.

**Table 1:** Item-wise analysis of knowledge test on scientific buffalo husbandry practices across subject areas.

**S. no**.	**Subject area**	**Item code**	**Item description (short)**	**Difficulty Index (*p*-value)**	**Corrected item-total correlation**	**Cronbach's alpha if item deleted**	**Status**
1	Feeding	F1	Dry matter % in buffalo feed	0.74	0.42	0.788	Selected
2	Feeding	F2	Frequency of mineral mixture use	0.68	0.39	0.791	Selected
3	Feeding	F3	Protein-rich fodder crop	0.72	0.33	0.796	Selected
4	Feeding	F4	Calcium:Phosphorus ratio	0.18	0.15	0.804	Rejected
5	Feeding	F5	Non-bypass protein source	0.88	0.1	0.807	Rejected
6	Breeding	B1	Optimal AI timing after estrus	0.65	0.41	0.785	Selected
7	Breeding	B2	Gestation period of buffalo	0.61	0.36	0.79	Selected
8	Breeding	B3	Number of registered buffalo breeds in India	0.58	0.4	0.782	Selected
9	Breeding	B4	Use of CIDR	0.22	0.16	0.799	Rejected
10	Breeding	B5	Dharwadi buffalo is the native of	0.85	0.12	0.808	Rejected
11	Health	H1	HS+BQ vaccine prevents which disease	0.71	0.47	0.774	Selected
12	Health	H2	Sign of subclinical mastitis	0.66	0.38	0.786	Selected
13	Health	H3	Deficiency responsible for retained placenta	0.63	0.35	0.789	Selected
14	Health	H4	Withdrawal period after antibiotic use	0.2	0.14	0.801	Rejected
15	Health	H5	Non-zoonotic disease among choices	0.87	0.1	0.806	Rejected
16	Milk Quality	MQ1	SNF value in buffalo milk	0.69	0.44	0.78	Selected
17	Milk quality	MQ2	Test to detect starch adulteration	0.65	0.4	0.785	Selected
18	Milk quality	MQ3	Permissible somatic cell count	0.71	0.36	0.786	Selected
19	Milk quality	MQ4	Enzyme indicating mastitis	0.19	0.1	0.807	Rejected
20	Milk quality	MQ5	Normal pH range of buffalo milk	0.89	0.09	0.81	Rejected
21	Milk marketing	MM1	Most common milk cooperative model	0.67	0.43	0.781	Selected
22	Milk marketing	MM2	Factor determining milk price	0.64	0.38	0.783	Selected
23	Milk marketing	MM3	Longest shelf-life milk product	0.7	0.41	0.778	Selected
24	Milk marketing	MM4	Product requiring cold chain	0.91	0.07	0.812	Rejected
25	Milk marketing	MM5	FSSAI's role in milk marketing	0.27	0.16	0.799	Rejected

Based on this criterion:

- Items with *p* < 0.30 (e.g., F4, B4, H4, MQ4, MM5; see Table 1 for descriptions) were considered too difficult and removed.
- Items with *p* > 0.90 (e.g., F5, B5, H5, MQ5, MM4; see Table 1 for descriptions) were considered too easy and removed.
- The remaining 15 items had *p*-values between 0.58 to 0.74, making them suitable for inclusion in the final test.

##### 3.4.1.2 Item discrimination

Item discrimination is the ability of a test item to differentiate between respondents with high and low levels of knowledge. It is quantified using point-biserial correlation between individual item scores and the total test score. The discrimination index ranges from −1 to +1, with values closer to +1 indicating strong discriminatory power. The criterion for point-biserial correlation values is as follows (27):

- >0.30: Good,
- 0.20–0.30: Acceptable,
- < 0.20: Poor; item should be revised or removed.

In this study, corrected item-total correlation values ranged between 0.09 and 0.47 as given in Table 1. Based on the criteria:

- Items with discrimination < 0.20 were dropped (e.g., F5, B5, H5, MQ5, MM4; see Table 1 for descriptions).
- Items with discrimination > 0.30 were retained.

After elimination, 15 items were retained with corrected item-total correlations between 0.33 and 0.47, indicating acceptable to good discrimination capacity.

##### 3.4.1.3 Internal consistency

Internal consistency refers to the reliability of the knowledge test. It was measured using Cronbach's alpha, which assesses the average correlation among items. A Cronbach's alpha value of 0.70 or above is generally considered acceptable for research purposes (28).

For the present 15-item scale the Cronbach's alpha = 0.792, indicating high internal consistency and none of the items, if deleted, reduced the overall reliability of the scale.

Thus, the final retained items were both reliable and well-structured as a composite measure.

##### 3.4.1.4 Validity

Content validity was ensured through expert consultation (27) with scientists specializing in buffalo husbandry at the ICAR-Central Institute for Research on Buffaloes (CIRB). Each item was carefully evaluated across five core thematic domains: Feeding, Breeding, Health, Milk Quality, and Milk Marketing. Experts reviewed the content, language, and relevance of each question in line with current scientific and practical practices.

### 3.5 Data collection

Data collection was integrated into the structured training programmes on scientific buffalo management practices conducted by ICAR-CIRB from January 2023 to December 2024. A standardized knowledge test consisting of 15 items, developed as part of the study instrument, was administered in two phases: a pre-training assessment to establish baseline knowledge levels, and a post-training assessment to measure immediate knowledge gains following the intervention. Both assessments were conducted in person at the training venues under uniform conditions to minimize external influences. The pre-test was administered on the first day prior to the delivery of any instructional content, while the post-test was conducted immediately after the conclusion of all training sessions. The same version of the test was used in both phases; however, in order to reduce recall bias, the sequence of the questions was shuffled in the post-test. The correct answers were not disclosed until the final day of the training, at which point they were discussed collectively with the participants. Furthermore, the purpose of the test was clearly explained at the outset to promote individual effort and discourage any form of copying or peer influence. Data collection was facilitated by trained field investigators and programme staff. For participants with limited literacy, enumerators read the questions aloud in the local language, clarified doubts without suggesting answers, and recorded responses verbatim. This inclusive approach enhanced the quality and accuracy of data, particularly among smallholder farmers from varied educational backgrounds. Alongside the knowledge assessments, a structured schedule was used to collect socio-economic information, including age, education level, landholding size, herd composition, and years of experience in buffalo rearing.

### 3.6 Data cleaning

All 530 interview schedules were comprehensively completed with no missing values. Mahalanobis distance, 1930 was computed to identify multivariate outliers using a *p*-value threshold of < 0.001 based on the chi-square distribution and degrees of freedom equal to the number of independent variables. This analysis identified 12 significant outliers (2.3% of the sample), which were removed, resulting in a final sample size of 518. The exclusion of these outliers did not materially affect the structural relationships or the overall findings of the study.

Mahalanobis distance was computed using the formula:


$$\begin{array}{c} D2=\;\left(x-\mu \right)TS-1\left(x-\mu \right) \end{array}$$

where x is the observed data vector, μ is the mean vector, and S−1 is the inverse covariance matrix of the indicators. Distances were compared to the chi-square distribution threshold for *p* < 0.001 with degrees of freedom equal to the number of indicators. Twelve responses exceeding the critical value were considered multivariate outliers and excluded, resulting in a final sample of 518.

### 3.7 Data analysis

A multi-tiered statistical approach examined the cognitive effectiveness of the training on scientific buffalo husbandry. Descriptive statistics provided a detailed profile of respondents across key socio-economic variables, including age, gender, education, herd size, and dairy experience. Cognitive improvement was analyzed using multiple indices—absolute gain, relative gain, percent gain, and Hake's normalized gain—capturing knowledge shifts from pre- to post-training phases. Group-wise comparisons by gender, livestock ownership, and experience levels revealed variations in knowledge enhancement across subpopulations. A multiple linear regression model identified socio-economic predictors significantly associated with cognitive gain, with variables such as education, herd size, experience, and pre-training knowledge score serving as key explanatory factors. Based on the model outputs, predicted gain scores were generated for different farmer profiles, offering insights into differential impacts of training. A chord diagram was developed to visualize interrelationships among gender, dairy experience, and levels of cognitive gain. Additionally, paired sample *t*-tests assessed within-group changes across the five core thematic components—feeding, breeding, health care, milk quality, and marketing—highlighting the significance of domain-specific learning improvements. To examine the domain-wise variation in cognitive improvement, a raincloud plot was generated to visualize the knowledge gain (post-test minus pre-test) across key subjects, including breeding, feeding, health, marketing, and milk quality. This composite visualization, combining boxplots, density plots, and jittered raw data points, provided an understanding of the distribution and magnitude of learning gains among participants.

## 4 Results

### 4.1 Socio-economic profile of the respondents

The socio-economic profile of the respondents of the study are given in Tables 2, 3. The average age of respondents was 40.9 years (SD = 13.8), reflecting a demographically mature population engaged in livestock farming. The gender distribution was relatively balanced, comprising 249 males (48.1%) and 269 females (51.9%). The respondents had an average of 9.98 years (SD = 5.45) of experience in dairy farming, suggesting moderate to substantial engagement in livestock-based livelihoods. Livestock ownership varied across the sample: 90 farmers (17.4%) owned 1–3 animals, 155 (29.9%) held 4–6, 138 (26.6%) maintained 7–9, and 135 (26.0%) owned 10 or more buffaloes. This variation in herd size indicates structural diversity within the farming community, which may have implications for adoption behavior, resource use efficiency, and access to services.

**Table 2:** Distribution of respondents by socio-economic categories (*n* = 518).

**Variable**	**Category**	**Count (percentage)**
Gender	
Male	249 (48.1%)
Female	269 (51.9%)
Livestock holdings			
1–3	90 (17.4%)
4–6	155 (29.9%)
7–9	138 (26.6%)
10+	135 (26.0%)

**Table 3:** Descriptive statistics of continuous socio-economic variables (*n* = 518).

**Variable**	**Mean**	**SD**	**Min**	**Max**
Age (years)	40.9	13.8	18	64
Dairy experience (years)	9.98	5.45	1	19

Model summary: R^2^ = 0.428. Adjusted R^2^ = 0.417. F_(5, 512)_ = 37.86. Model Significance: < 0.001^***^.

### 4.2 Measuring cognitive gain

The cognitive gain of the respondents was assessed using four metrics: absolute gain, relative gain, Hake's normalized gain, and percent gain as given in Table 4. These indicators collectively provide a robust understanding of the knowledge improvement resulting from the training intervention. The absolute gain, representing the simple difference between post-test and pre-test scores, was found to be 4.63 (SD = 2.28). This indicates that, on average, respondents improved their scores by ~4.6 points after the training, suggesting a noticeable knowledge acquisition across the group. The relative gain, calculated as the improvement in score relative to the pre-test score, was 0.65 (SD = 0.52). This signifies that respondents, on average, achieved a 65% improvement over their initial knowledge level, reflecting the effectiveness of the program especially for those with lower baseline scores. The Hake's normalized gain, which standardizes the gain based on the maximum possible improvement, was 0.70 (SD = 0.28). According to Hake's criterion, a normalized gain of >0.7 is considered high, 0.3–0.7 as moderate, and < 0.3 as low. The value of 0.70 observed in this study places the knowledge gain at the upper threshold of moderate, suggesting that the intervention was pedagogically effective and significantly enhanced cognitive understanding. The percent gain, representing the gain as a percentage of the total maximum possible score, was 30.8% (SD = 15.2%). This again affirms a substantial increase in knowledge levels attributable to the training. Therefore, the cognitive gain metrics reveal a consistent pattern of significant improvement, with relatively low variability. These results affirm the efficacy of the capacity-building intervention in enhancing the cognitive competencies of smallholder buffalo farmers in the study area.

**Table 4:** Summary of cognitive gain measures among respondents (*n* = 518).

**Gain type**	**Mean**	**SD**
Absolute gain	4.63	2.28
Relative gain	0.65	0.52
Hake's normalized gain	0.7	0.28
Percent gain	30.8	15.2

In order to explore patterns in learning outcomes, cognitive gains were disaggregated across gender, experience levels, and landholding categories.

#### 4.2.1 Gender-wise cognitive gain

**Figure 3:**
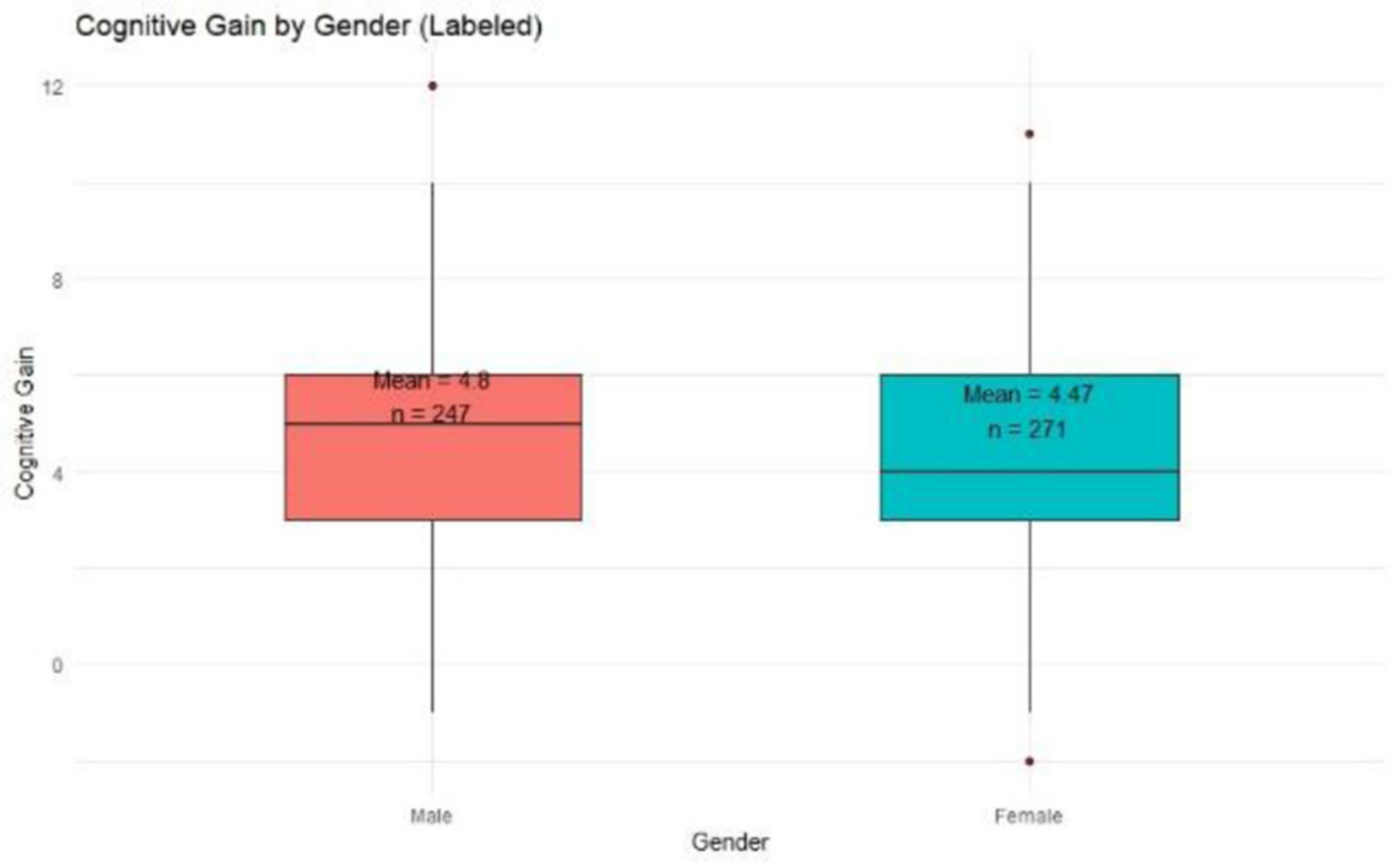
Cognitive gain by gender.

The analysis (Figure 3) revealed that male respondents demonstrated a slightly higher average cognitive gain (Mean = 4.80) compared to their female counterparts (Mean = 4.47). Both groups exhibited similar interquartile ranges (Q1 = 3, Q3 = 6), indicating consistent knowledge acquisition. The median gain was higher among males (Median = 5), suggesting better post-training comprehension.

#### 4.2.2 Experience group-wise cognitive gain

**Figure 4:**
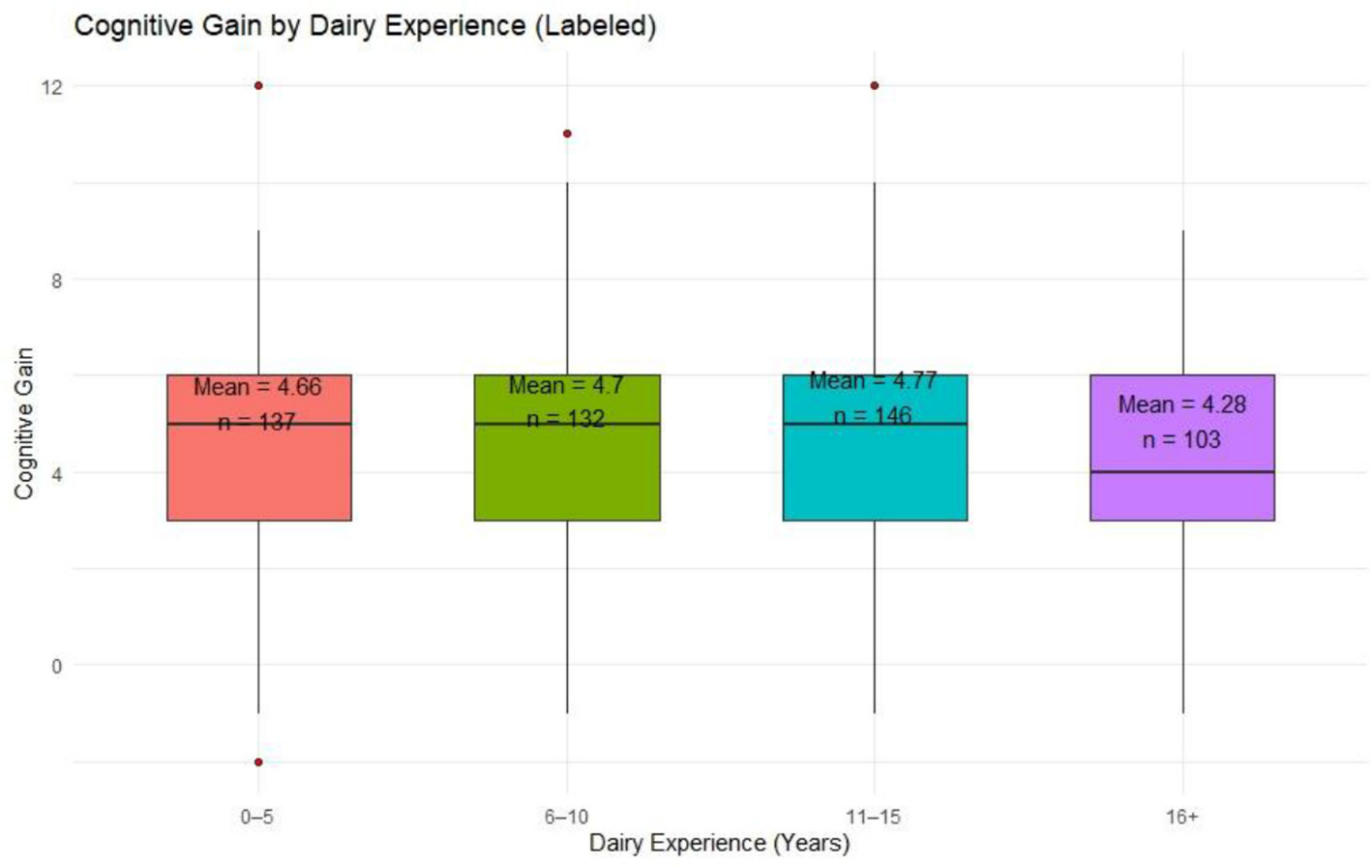
Cognitive gain by dairy experience.

Cognitive gain varied modestly across experience groups (Figure 4). Participants with 11–15 years of dairy experience recorded the highest mean gain (Mean = 4.77), followed by those with 6–10 years (Mean = 4.70) and 0–5 years (Mean = 4.66). Interestingly, respondents with over 16 years of experience had the lowest mean gain (Mean = 4.28), implying a potential plateau or resistance to new learning among the most experienced farmers. However, all groups shared similar medians (Median = 4–5) and interquartile ranges, pointing to overall consistency.

#### 4.2.3 Holding group-wise cognitive gain

**Figure 5:**
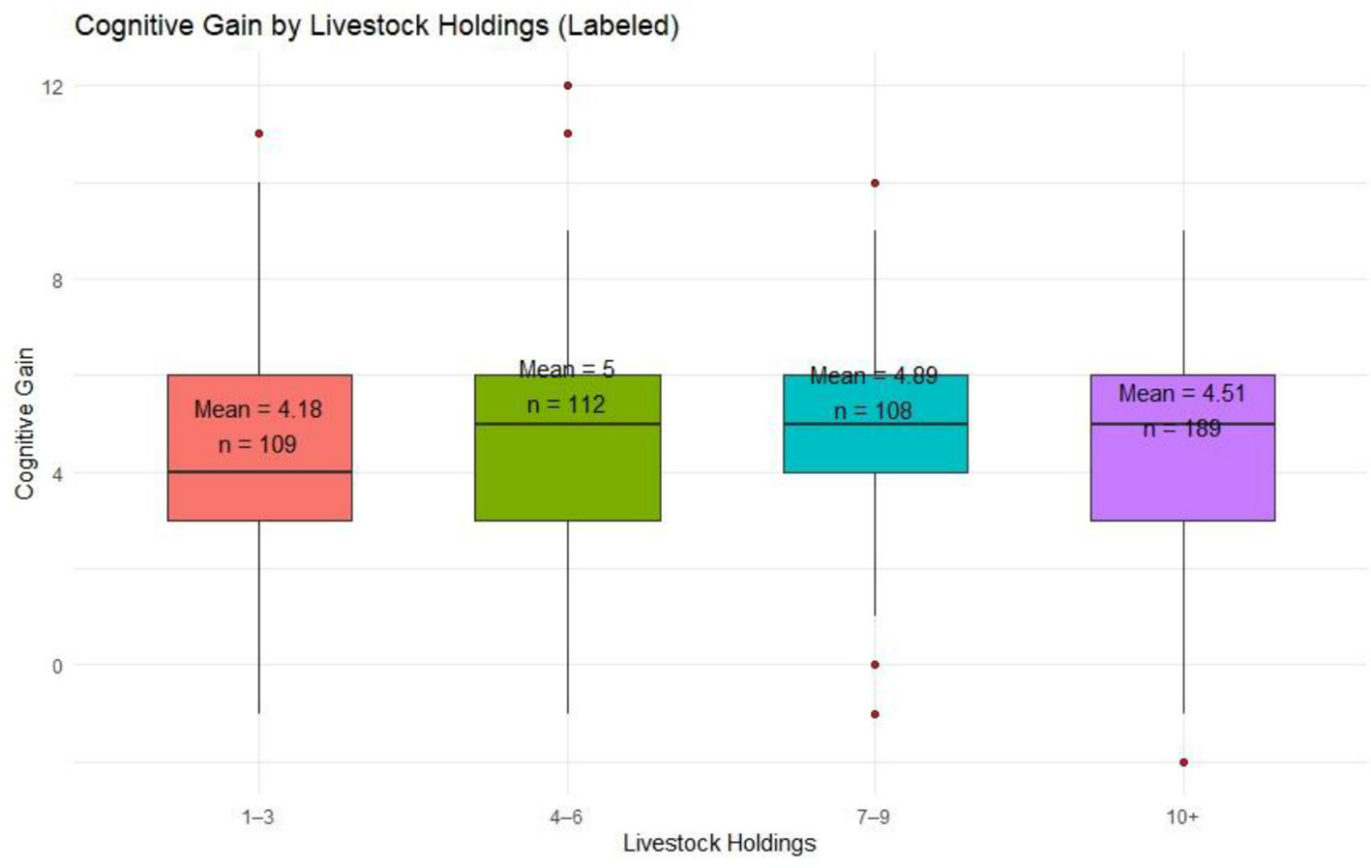
Cognitive gain by livestock holdings.

When analyzed by landholding size (Figure 5), respondents with 4–6 acres showed the highest average gain (Mean = 5.00), followed by those with 7–9 acres (Mean = 4.89). Farmers with the smallest holdings (1–3 acres) and the largest (10+ acres) reported lower mean gains (4.18 and 4.51, respectively). The disaggregated analysis demonstrates that gender, experience, and landholding size subtly influence the magnitude of cognitive improvement. These insights can inform the design of more tailored extension strategies targeting specific subgroups within the farming community.

### 4.3 Determinants of cognitive gain among smallholder buffalo farmers

The multiple linear regression model was employed to identify key predictors of cognitive gain among smallholder buffalo farmers (*n* = 518) as given in Table 5. The model explained 42.8% of the variance in cognitive gain, as indicated by an R-squared (R^2^) value of 0.428, with an adjusted R^2^ of 0.417, demonstrating a good model fit. The overall model was statistically significant [F_(8, 509)_ = 37.86, *p* < 0.001], indicating that the set of predictors reliably explains variation in cognitive gain.

**Table 5:** Multiple linear regression predicting cognitive gain (*n* = 518).

**Predictor**	**Estimate β)**	**Std. error**	***t*-value**	***p*-value**
Intercept	2.8	0.32	8.75	< 0.001^***^
Gender (female)	0.52	0.16	3.25	0.001^**^
Age (years)	−0.02	0.008	−2.50	0.013^*^
Dairy experience (yrs)	0.05	0.014	3.57	< 0.001^***^
Livestock holdings	0.08	0.022	3.64	< 0.001^***^
Pre-test score	−0.31	0.05	−6.20	< 0.001^***^

Model summary: R^2^ = 0.428. Adjusted R^2^ = 0.417. F_(5, 512)_ = 37.86. Model Significance: < 0.001^***^.

Among the significant predictors, gender showed a positive effect, with male respondents gaining 0.52 points more on average than females (*p* = 0.001), suggesting greater receptivity or engagement among female during the training. Age had a small but significant negative association (β = −0.02, *p* = 0.013), indicating a slight decline in cognitive gain with increasing age. In contrast, dairy farming experience was positively associated with cognitive improvement (β = 0.05, *p* < 0.001), suggesting that practical exposure reinforces conceptual understanding during training sessions. Livestock holdings also had a positive effect (β = 0.08, *p* < 0.001), implying that farmers with larger herd sizes may be more motivated or better positioned to apply new knowledge. Importantly, the pre-test score was a strong negative predictor of cognitive gain (β = −0.31, *p* < 0.001), likely due to a ceiling effect—those who began with higher knowledge had less room for improvement.

### 4.4 Predicted cognitive gain across socio-demographic groups

**Figure 6:**
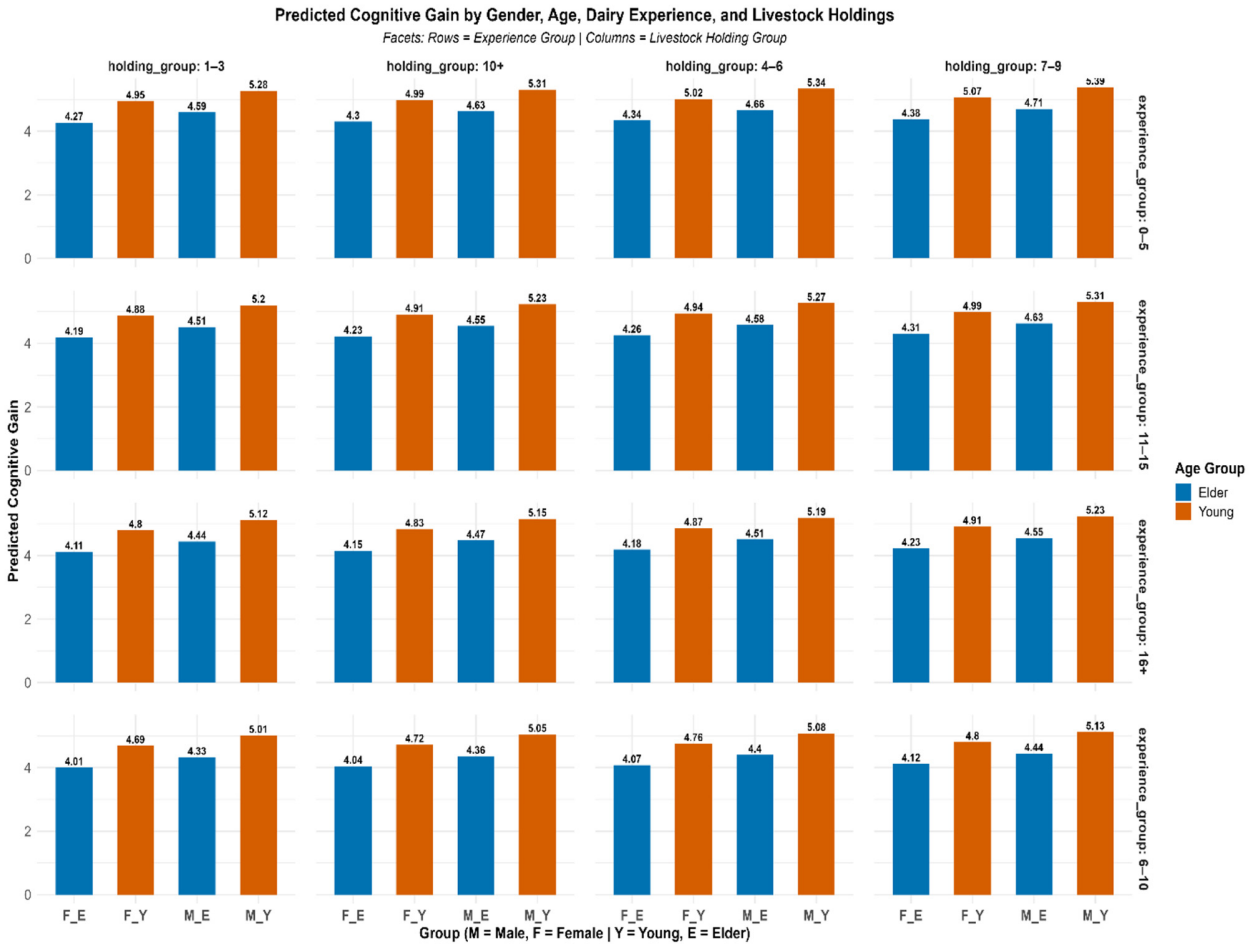
Predicted cognitive gain by socio-economic variables.

To further elucidate the impact of key socio-demographic factors on cognitive gain, predicted values were generated using the fitted multiple linear regression model. These predictions were stratified across combinations of gender, age group, dairy farming experience, and livestock holding size as indicated in Figure 6. A consistent pattern emerged, wherein young female farmers with higher livestock holdings demonstrated the highest predicted cognitive gains. For example, young females with 0–5 years of experience and a holding size of 7–9 animals had a predicted gain of 5.07, compared to 4.38 for their elder counterparts under identical conditions. Similarly, young males consistently outperformed elder males, with the highest predicted gain (5.39) observed among young males having 0–5 years of experience and 7–9 animals. Across all experience brackets (0–5, 6–10, 11–15, and 16+ years), the gender and age interactions remained prominent—younger respondents, particularly females, exhibited greater learning gains. This suggests that youth and female engagement in training programs may be associated with enhanced receptivity or application of knowledge. Livestock holding size also played a reinforcing role, as predicted gains increased with herd size, likely reflecting greater motivation to utilize new knowledge in a larger-scale operation. These group-wise predicted gains provide further support to the regression model findings and highlight the importance of tailoring capacity-building interventions to target young and female farmers with growing herd sizes to maximize cognitive impact.

### 4.5 Mapping the interplay of gender and experience with cognitive gain categories

**Figure 7:**
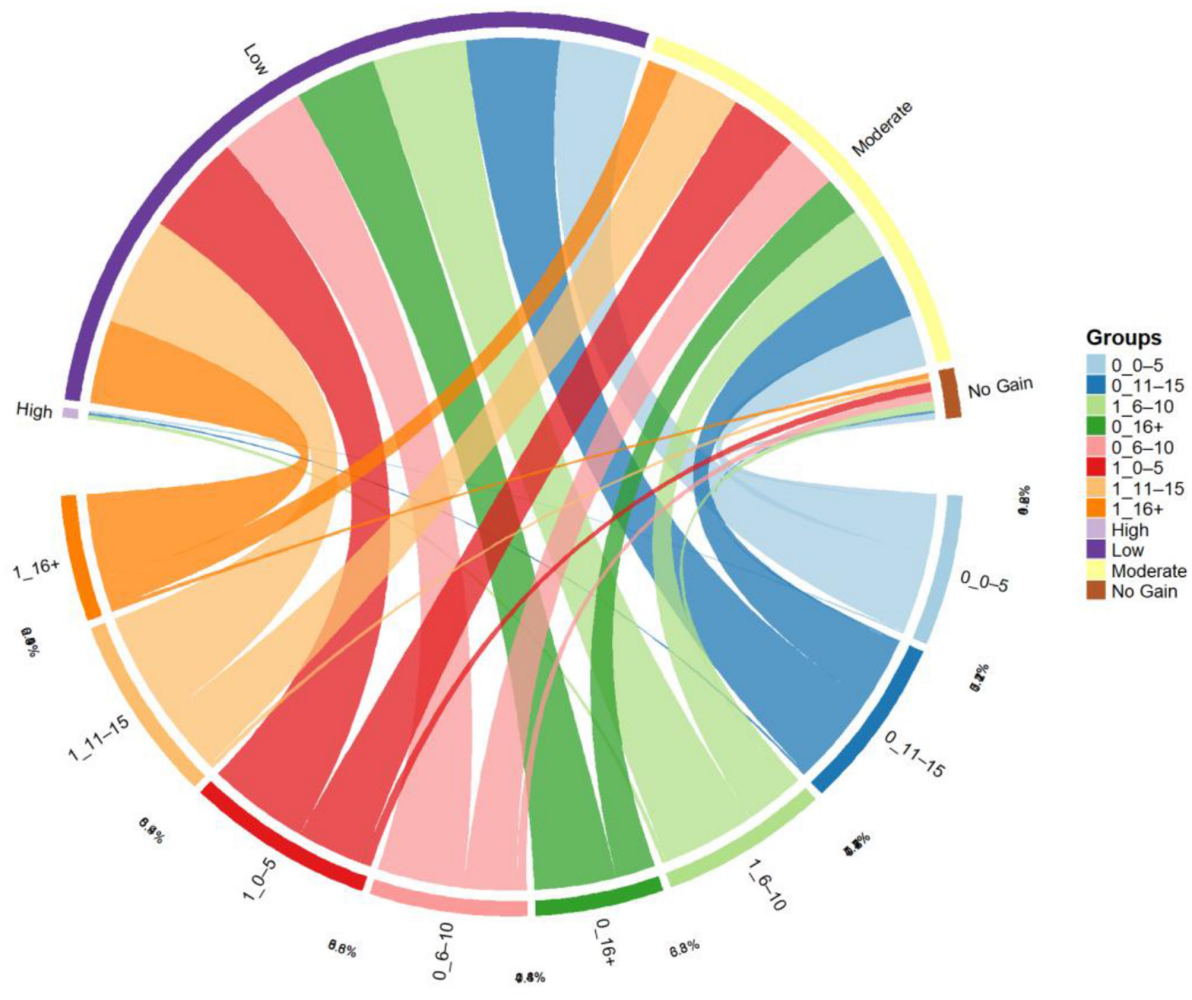
Chord diagram depicting cognitive gain distribution by gender and experience.

The chord diagram (Figure 7) visually represents the association between gender-experience categories (Source_Group) and levels of cognitive gain (High, Moderate, Low, and No Gain). The classification follows a combined coding format (e.g., 1_6–10), where the first digit denotes gender (1 = Female, 0 = Male), and the second denotes years of dairy experience. A notable pattern observed is the predominance of Low Gain across both genders and experience levels, accounting for a substantial proportion of the total connections. Among females with 11–15 years of experience (1_11–15), the highest frequency of Low Gain (8.9%) was recorded, followed closely by 1_0–5 (8.3%) and 1_6–10 (7.7%), indicating that even experienced women did not always translate prior exposure into higher knowledge acquisition. Interestingly, Moderate Gain was also visible across all groups, particularly among females in the 1_0–5 (5.6%) and 1_11–15 (5.4%) categories, as well as males in 0_11–15 (5.4%) and 0_6–10 (4.4%), suggesting a consistent moderate-level improvement regardless of gender. However, High Gain was relatively rare and concentrated within select profiles—specifically, females with 6–10 years of experience (1_6–10) and males with either 0–5 or 11–15 years of experience (0_0–5, 0_11–15)—each registering only 0.2–0.4% of the sample. Conversely, No Gain outcomes, though infrequent, were dispersed across multiple categories, including both males and females with < 15 years of experience. This dispersion reflects underlying heterogeneity in knowledge absorption that may stem from factors such as prior exposure, motivation, or delivery methods of training. Hence it is evident from the diagram that while cognitive gains were evident across all strata, the magnitude of gain was more consistently moderate, with fewer instances of transformative learning. Gender and experience interplay in shaping learning outcomes, but are not deterministic in isolation—highlighting the need for tailored extension strategies that consider diversed learner profiles.

*The chord diagram presents the flow between predictor groups (e.g., gender, education, livestock holding, training intensity, baseline knowledge) and levels of cognitive gain (No Gain, Low, Moderate, High). Each colored segment on the circumference represents a category within these variables. The connecting ribbons (chords) indicate the proportion of respondents transitioning from each predictor category to a specific cognitive gain level. Thicker chords represent stronger associations or larger proportions. For example, a prominent flow from the ‘High” baseline knowledge group to the “Low” gain group suggests limited improvement in already knowledgeable participants. Conversely, substantial flows from “Low” or “Moderate” categories toward “High Gain” reflect effective training impact on underprepared participants*.

### 4.6 Differential cognitive gains across subject areas

**Figure 8:**
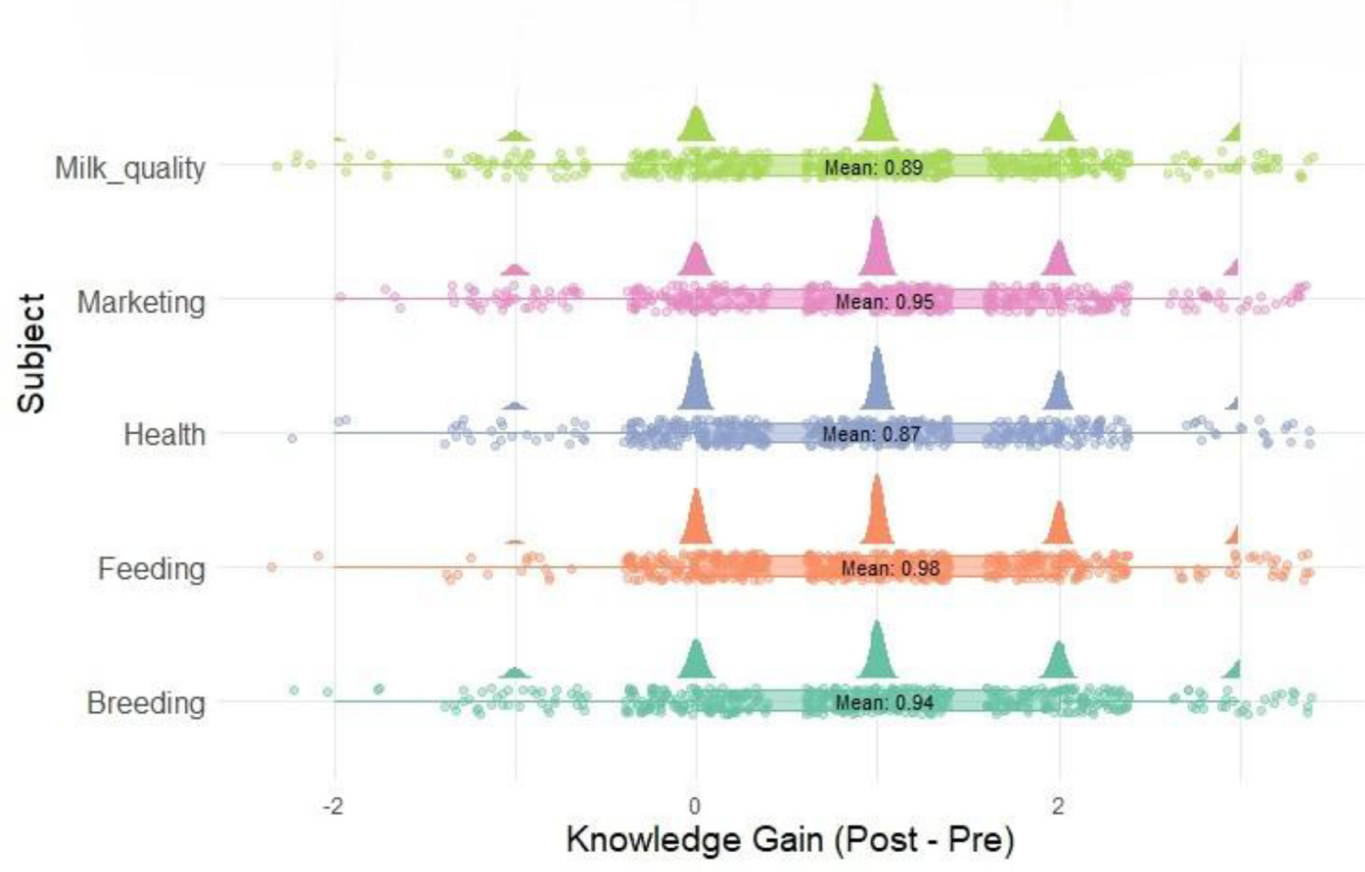
Raincloud plot of knowledge gain by subject.

An in-depth subject-wise analysis of cognitive gain (Figure 8) revealed that respondents demonstrated statistically significant improvements across all five thematic domains of the knowledge test, with *p*-values consistently below 0.001. However, the magnitude of knowledge acquisition varied by subject area, reflecting differential responsiveness to the training intervention.

The Feeding domain recorded the highest mean gain (Mean = 0.977, t = 23.42), suggesting that farmers were highly receptive to new insights on feed management, ration balancing, and mineral supplementation. This may be attributed to the direct and visible impact of feeding practices on animal productivity, particularly milk yield, which aligns closely with farmers' livelihood priorities. Moreover, smallholder farmers often lack formal training in nutritional planning, which may have led to a larger knowledge deficit—and consequently, greater gain when exposed to scientifically sound feeding strategies. Milk Marketing (Mean = 0.948) and Breeding (Mean = 0.944) also exhibited high gains. In the case of milk marketing, the cognitive improvement could stem from the novelty of the concepts shared, such as pricing strategies, hygiene in milk collection, and cooperative-based selling models—topics generally underrepresented in routine extension interactions. Similarly, the emphasis on AI timing, record-keeping, and sire selection in the breeding module may have resonated strongly with participants, particularly as these areas are critical to reproductive efficiency but often misunderstood or misapplied in traditional systems. The domains of Milk Quality (Mean = 0.886) and Animal Health (Mean = 0.873) showed relatively lower—yet still significant—cognitive gains. This may reflect a higher baseline familiarity with basic health practices such as deworming, mastitis control, or cleanliness, which are commonly disseminated via veterinary outreach programs. Additionally, certain health topics covered—such as subclinical infections or immunization protocols—may have been perceived as technically complex, limiting immediate assimilation despite training efforts. The variation in cognitive gain highlights the importance of tailoring training content to the existing knowledge base and contextual needs of learners. It also suggests that practical, outcome-oriented subjects (e.g., feeding and marketing) tend to elicit higher engagement and retention among adult learners in livestock-based livelihoods.

## 5 Discussion

This study examined the cognitive effectiveness of a structured training intervention aimed at enhancing farmers' knowledge of scientific buffalo husbandry in India. The study assessed how training content impacted farmers' understanding across five key domains: feeding, breeding, health, milk quality, and milk marketing by employing a pre-test–post-test design grounded in adult learning theory. The discussion below interprets these findings in relation to educational theory, subject-specific trends in knowledge improvement, and socio-economic factors that influenced learning outcomes. These insights are intended to inform future veterinary extension practices and strengthen training programme design for smallholder dairy systems.

Veterinary education plays a pivotal role in strengthening livestock production systems, particularly in resource-constrained settings where the burden of animal health management often rests with farmers. This study directly contributes to veterinary science by evaluating the cognitive outcomes of a structured veterinary education programme designed to improve scientific buffalo husbandry practices among smallholder dairy producers. In the Indian context—where buffalo-based dairy systems form the backbone of rural livelihoods and nutrition security—enhancing farmers' understanding of veterinary principles such as disease prevention, hygienic milk production, reproductive management, and nutrition is essential for improving herd health, milk quality, and farm profitability.

Unlike conventional evaluations that rely primarily on subjective feedback or attendance metrics (29–31), this study employed a quasi-experimental pre-test–post-test design using a psychometrically validated instrument to objectively assess knowledge acquisition. This methodological approach is consistent with the emerging emphasis on evidence-based veterinary extension and provides actionable insights into how structured capacity-building interventions can contribute to improving veterinary literacy among farming communities (32).The theoretical underpinning of this study draws from adult learning theory, which highlights the self-directed nature of adult learners and emphasizes the relevance, practicality, and experience-based integration of new knowledge (24). These principles are particularly applicable to smallholder buffalo farmers, whose knowledge is often experiential but lacks alignment with current scientific veterinary practices. The substantial cognitive gains observed in practical domains—such as feeding and milk marketing—demonstrate the effectiveness of aligning training content with immediate livelihood challenges and contextual realities (33–35). These findings also support the broader pedagogical understanding that adult learners retain knowledge more effectively when the material is closely linked to their routine decision-making processes (36, 37). However, relatively lower knowledge improvement in modules related to animal health and milk quality emphasizes an important educational barrier. These topics often encompass conceptually dense veterinary content, including disease identification, vaccination schedules, subclinical mastitis management, and hygienic milking procedures—areas which demand greater cognitive effort and prior exposure. Cognitive Load Theory offers a valuable explanation, suggesting that when information is too complex or insufficiently scaffolded, it can overwhelm the learner's capacity to assimilate and retain it (38–40). This implies the need for more structured instructional strategies in veterinary extension, such as using infographics, hands-on demonstrations, visual simulations, and follow-up reinforcement to ensure improved knowledge transfer in these domains (7, 8, 10, 13).

In addition to developing a structured training intervention, this study introduced an empirically validated knowledge test, covering core domains of buffalo husbandry, as a tool for objectively evaluating programme impact. A multi-metric assessment approach—combining absolute, relative, and normalized gain indices—was used to evaluate learning outcomes as objective cognitive improvement, offering a deeper understanding of training effectiveness beyond subjective participant feedback. The use of such metrics strengthens the evidence base for veterinary training design and enables preliminary evaluation of the educational value delivered. While a direct return on investment cannot be inferred without assessing the practical application of knowledge, these indicators offer a foundational understanding of training effectiveness. The study also identified key socio-demographic variables—such as gender, age, herd size, and dairy farming experience—that significantly influenced knowledge improvement. A particularly noteworthy finding was the negative correlation between baseline knowledge and post-training gain, suggesting a potential ceiling effect. This aligns with adult learning theory, which emphasizes that adults are more receptive to learning experiences that are novel, relevant, and tailored to their prior experiences. Participants with higher baseline knowledge may have encountered limited new material, resulting in lower cognitive gains. In contrast, those with moderate experience and larger herd sizes appeared to derive greater value, likely because they found stronger personal and contextual relevance in the content. Adult learning principles suggest that when learners perceive relevance and are treated as co-creators of knowledge, their motivation to engage increases, enhancing the likelihood of meaningful knowledge acquisition (25, 26). Participants entering the training with high baseline knowledge may have found less novelty or challenge in the material, leading to limited cognitive gain. Conversely, those with moderate experience likely encountered more meaningful learning opportunities, resulting in greater knowledge acquisition. This pattern highlights the importance of aligning training content with learners' prior knowledge and experience levels to maximize educational effectiveness.

In contrast, moderate experience levels and larger herd sizes were associated with greater knowledge acquisition, suggesting higher intrinsic motivation and stronger perceived relevance of the training. These insights can guide veterinary educators and extension planners in targeting high-impact groups—such as young female farmers with moderate experience—for future interventions. The subject-wise analysis revealed that training in feeding, breeding, and milk marketing produced the most substantial cognitive gains. These areas are often underrepresented in traditional veterinary extension but offer high visibility and immediate benefit to farmers (10, 13). In contrast, animal health and milk quality—despite being central to veterinary science and public health—demonstrated limited cognitive gains, reinforcing the need for veterinary-specific pedagogical refinement(10). Incorporating simplified diagnostic charts, decision trees, and participatory group discussions may enhance comprehension in such technically demanding areas (13). This study also contributes to the broader veterinary discourse by reinforcing the relevance of veterinary extension in achieving public health and One Health goals such as such as controlling zoonotic diseases, improving food safety, and promoting antimicrobial stewardship (12). Improving farmers' knowledge in areas such as zoonotic disease prevention, antimicrobial stewardship, and hygienic animal handling enhances animal productivity, food safety, and community health, aligning with the core objectives of the One Health framework (13). The findings highlight the importance of integrating veterinary principles within training design and evaluation frameworks and advocate for structured, measurable, and adaptive approaches to veterinary education at the grassroots level. The study offers a replicable model for evaluating the effectiveness of veterinary training interventions in livestock systems. It advances both the pedagogical and technical dimensions of farmer capacity building by combining educational theory with veterinary content. The study affirms the value of integrating cognitive outcome evaluation in veterinary extension and provides critical insights for designing more effective, targeted, and scalable training programmes that can improve animal health, milk quality, and rural resilience.

## 6 Conclusion

This study reaffirms the growing importance of evidence-based veterinary extension as a cornerstone of livestock development, particularly within the context of smallholder buffalo husbandry in India. It offers a diagnostic lens to evaluate how effectively veterinary knowledge—spanning disease prevention, reproductive health, nutrition, and hygienic milking—is internalized by livestock farmers by integrating the principles of adult learning with structured cognitive assessment. Rather than viewing training as a static information transfer mechanism, the study conceptualizes it as a learner-mediated and context-driven process, wherein cognitive improvement is not merely a knowledge outcome but a proxy for veterinary service outreach effectiveness. Importantly, this research expands the understanding of veterinary education beyond technical dissemination. It situates training as a transformative platform for behavioral recalibration, enabling farmers to adopt practices aligned with core veterinary objectives—such as mastitis prevention, vaccination adherence, rational use of anthelmintic and antimicrobials, and safe milk production. These shifts hold direct implications for improving animal health outcomes, reducing production losses, enhancing milk quality, and ultimately protecting public health through better zoonosis control and antimicrobial resistance (AMR) mitigation. The study also highlights the value of tailoring veterinary extension to accommodate learner heterogeneity—recognizing that variables such as gender, education, and herd size critically influence receptivity to veterinary knowledge. This perspective calls for a departure from traditional one-size-fits-all approaches and encourages the development of adaptive, modular veterinary training frameworks, capable of responding to localized epidemiological profiles and farmer realities. The study contributes a replicable framework for improving accountability, targeting, and impact assessment in capacity-building programmes by operationalizing cognitive gain as a measurable output of veterinary extension. The insights generated support the institutionalization of performance-based veterinary education models, where learning outcomes guide not only training refinement but also resource allocation within schemes such as the National Livestock Mission and Rashtriya Gokul Mission (13). The research offers a compelling case for transforming veterinary extension into an outcome-oriented, learner-sensitive, and behaviorally grounded system. It lays the groundwork for a new generation of livestock advisory models—where veterinary science is not limited to clinical services but embedded within a broader pedagogical and development agenda. Such models are essential for achieving sustainable livestock production, ensuring animal welfare, and safeguarding public health in the face of evolving challenges such as climate stress, emerging diseases, and AMR.

## 7 Policy implications

### 7.1 Mandatory integration of cognitive assessment in veterinary training programs

Establishing baseline and post-training knowledge evaluation as a mandatory protocol across veterinary and livestock training programs can help quantify learning outcomes and identify critical knowledge gaps. Making such evaluations a standard practice under centrally sponsored schemes like the Rashtriya Gokul Mission (RGM) and National Livestock Mission (NLM) can enhance accountability and training effectiveness.

### 7.2 Designing region-specific and practice-oriented curricula

Developing curricula tailored to regional livestock profiles, especially buffalo-based production systems, can address the observed variation in knowledge gain across subjects. Emphasizing practical demonstrations, indigenous practices, and localized challenges within course content will likely improve knowledge retention and relevance among smallholder farmers.

### 7.3 Incorporating gender-inclusive training strategies within extension systems

Institutional frameworks need to formally incorporate gender-responsive training approaches. Adapting module delivery for women—considering time constraints, mobility, literacy, and household responsibilities—can improve access, comprehension, and participation. Leveraging community-based female trainers and peer-led models can further increase engagement.

### 7.4 Linking funding disbursement with measurable learning outcomes

Shifting from input-based to outcome-linked disbursement of training funds can incentivize performance in veterinary extension services. Establishing performance metrics based on cognitive gain, alongside participation and behavioral change indicators, will enable funding bodies to prioritize impactful training modules.

### 7.5 Embedding behavioral science principles into veterinary extension programs

Training designs should incorporate elements like social proof, reinforcement through nudges, and culturally relevant motivational triggers. Addressing KAP gaps requires more than knowledge transfer—relying on behavioral change models can improve practice adoption among farmers.

### 7.6 Developing a national evaluation framework for veterinary capacity-building initiatives

Introducing a standardized evaluation toolkit across state departments, Krishi Vigyan Kendras (KVKs), and livestock training institutes can ensure systematic tracking of training impacts. Such a framework should include pre-post assessments, learner feedback, and context-specific quality indicators.

### 7.7 Prioritizing training programs for high-yielding but poorly informed segments

Households engaged in buffalo husbandry, often contributing significantly to national milk production yet showing persistent KAP gaps, deserve priority in future training outreach. Tailoring extension programs to these segments can address inefficiencies in knowledge-to-practice translation and enhance productivity sustainably.

## 8 Limitations of the study

This study was designed to evaluate immediate cognitive changes among trainees following structured capacity-building sessions in scientific buffalo husbandry. Given this scope, certain aspects such as long-term behavioral transformation, practical adoption, and productivity gains were not included. The absence of a control group limits direct attribution of knowledge gains solely to the intervention; however, the use of a quasi-experimental pre-post design still allowed for meaningful inference of training effectiveness. The voluntary participation of learners may reflect a self-selection effect, but this also mirrors real-world program outreach, where training is often accessed by motivated individuals. These parameters were aligned with the study's intent and timeline, and do not undermine the value of the results. Instead, they open avenues for future inquiry.

## 9 Future research

Building upon the insights of this study, the following directions are proposed to extend and deepen the research on training effectiveness in livestock-based extension systems:

### 9.1 Adoption of longitudinal evaluation designs

While the present study successfully measured immediate cognitive gains, it does not capture long-term retention or actual behavioral transformation. Future research should adopt longitudinal tracking methods to assess how acquired knowledge translates into on-farm practices over time, thereby bridging the gap between learning and sustained impact.

### 9.2 Integration of behavioral and performance metrics

Although cognitive gain is a critical first step, it is only one dimension of training effectiveness. Future studies should incorporate behavioral observations and productivity indicators—such as changes in herd health, milk yield, or reproductive efficiency—to assess whether learning outcomes lead to measurable improvements in livelihood performance.

### 9.3 Incorporation of psychometric and motivational constructs

Exploring learner-related variables such as self-efficacy, intent to change, perceived utility, and motivational readiness can improve the explanatory power of training evaluation models. The inclusion of behavioral theories, such as the Theory of Planned Behavior, may enrich understanding of how knowledge transitions into action.

### 9.4 Application of mixed methods and contextual analysis

Veterinary training often takes place within complex socio-cultural and agro-ecological environments. Mixed-method research—combining quantitative assessments of knowledge/performance with qualitative insights from farmer interviews, focus groups, or ethnographic observation—can reveal contextual barriers to adoption, such as traditional norms, mistrust in veterinary systems, or logistical challenges in accessing services. Such insights would be invaluable for tailoring veterinary extension strategies to local needs and constraints.

### 9.5 Evaluation of digital and blended veterinary learning platforms

With the rise of digital agriculture, future research should assess the effectiveness of ICT-enabled, mobile-based, or blended learning modules in veterinary education. Evaluating the cognitive, behavioral, and clinical outcomes of such platforms, especially among women and geographically isolated livestock keepers, will help determine their viability as scalable alternatives to in-person training.

## Data Availability

The raw data supporting the conclusions of this article will be made available by the authors, without undue reservation.
